
JOURNAL INFORMATION
==============================
NLM Title Abbreviation: ScientificWorldJournal
Journal ID: ScientificWorldJournal
NLM Journal ID: 101131163
Journal ID: TSWJ

The Scientific World Journal

ISSN: 2356-6140
EISSN: 1537-744X
Publisher: Wiley

ARTICLE INFORMATION
==============================
PMCID: PMC5956491
PMID: 29861677
DOI: 10.1100/tsw.2004.78
Article ID: 210475
Article version: 1
Subjects: Original Report

Prune Belly Syndrome and Tracheo-esophageal Fistula in a Premature Neonate

Potter Lance 1
Rosser Charles J. 1
Iskandar S. 2
Kroovand R. Lawrence 1
1 Departments of Urology, Wake Forest University School of Medicine, Winston-Salem, North Carolina 27157, USA
2 Departments of Pathology, Wake Forest University School of Medicine, Winston-Salem, North Carolina 27157, USA
Academic Editor: Anthony Atala

Publication date: 2004
Electronic publication date: 2004 Jun 7
Volume: 4
First page: 291
Last page: 295
Copyright: Copyright © 2004 Lance Potter et al.
Copyright year: 2004
License: This is an open access article distributed under the Creative Commons Attribution License, which permits unrestricted use, distribution, and reproduction in any medium, provided the original work is properly cited.
License URL: https://creativecommons.org/licenses/by/3.0/

==============================
Original Report
*TheScientificWorldJOURNAL* (2004) 4 (S1), 291–295
ISSN 1537-744X; DOI 10.1100/tsw.2004.78

### Prune Belly Syndrome and Tracheo-esophageal Fistula in a Premature Neonate

Lance Potter[1], Charles J. Rosser[1], S. Iskandar[2], and R. Lawrence Kroovand[1]
*Departments of Urology[1] and Pathology[2], Wake Forest University School of Medicine, Winston-Salem, North Carolina 27157*

*Previously published in the Digital Urology Journal*

**DOMAIN**: urology

Prune Belly Syndrome, also known as Eagle-Barrett syndrome, is a well known triad of abdominal muscle deficiency or hypoplasia, urinary tract anomalies, and bilateral cryptorchidism.[1] Tracheo-esophageal fistula is a sporadically occurring defect estimated to occur in 1 out 4425 live births.[2] Only one case of Prune Belly Syndrome, tracheo-esophageal fistula associated with VATER syndrome, and urethral atresia occurring in the same individual has been reported.[3] We present a unique case involving a 33 week fetus with bladder distention and bilateral hydroureteronephrosis diagnosed in utero who postnatally was diagnosed with Prune Belly Syndrome, tracheo-esophageal fistula, and urethral atresia.

### CASE REPORT

The patient is a twin female product of a diamniotic, monochorionic gestation. Prenatal ultrasound at nineteen weeks showed marked bladder distention and bilateral hydroureteronephrosis. Shortly thereafter in utero, the patient underwent weekly bladder drainage, removing over 2000 milliliters over the next thirteen weeks. During this time, a vesico-amniotic shunt was unsuccessfully placed due to anatomically problems. Amniocentesis prior to delivery showed normal 46 XX chromosomes. The patient was born at 33 weeks via spontaneous vaginal delivery to a 24 year old white female with no known medical diseases. Respiratory failure ensued requiring ventilatory support. The baby's initial physical examination was significant for Potter's facies, decreased breath sounds bilaterally, and a continuous heart murmur. She was also noted to have wrinkled skin over a lax abdominal wall with easily palpable internal organs and large bilateral flank masses. Her genitourinary exam revealed moderate clitoromegaly with scrotal/labial folds but no identifiable urethral meatus or vaginal vestibule. Rectal exam revealed a normal positioned anus with a paucity of tone.

After the placement of a 8 Fr. percutaneous suprapubic tube, a cystogram was performed which demonstrated a large bladder with severe trabeculation. After instillation of 200 milliliters of cystograffin under gravity, bilateral grade 5 ureteral reflux was evident by the presence of contrast in the renal pelvis bilaterally (Figure1). Next, abdominal ultrasonography was performed revealing moderate bilateral hydroureteronephrosis, thickened bladder wall, and free intraperitoneal fluid. The patient's initial laboratory results revealed a normal serum creatinine of 0.3 gm/dl. Her postnatal course was complicated

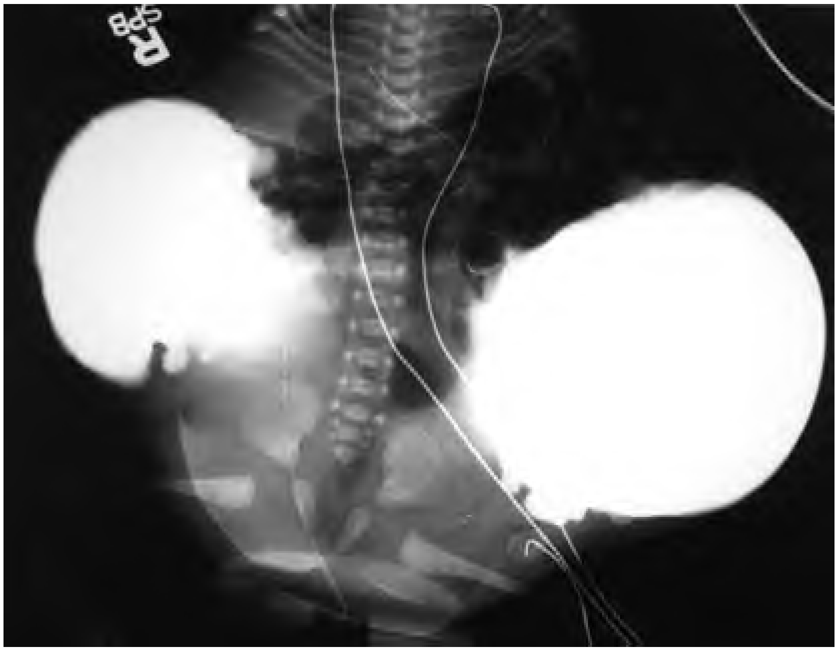

**FIGURE 1**. Cystogram demonstrating severe bilateral reflux of contrast into renal pelvis bilaterally.

by the discovery of free intraabdominal air on day 1 of life. The patient was taken immediately to the operating room where a gastric perforation was discovered secondary to a distal tracheo-esophageal fistula with aproximal pouch. No esophageal atresia was present. The esophagus was ligated and a gastrotomy tube placed.

On day four of life, the patient was noted to have a creatinine of 1.0mg/dl. Urine output during this time was between 1.5-3 ml/kg/hr. Repeat abdominal ultrasonography demonstrated worsening hydroureteronephrosis. The patient was taken to the operating room for an exploratory laparotomy and urinary diversion. The bladder was noted to have two large diverticuli emanating each from the postero-lateral aspect of the bladder with a dilated urachal remnant. (Figure2) A transverse incision was used to open the bladder which was noted to be severely thickened and trabeculated. The right ureteral orifice was seen draining into the right diverticulum while the left ureteral orifice was identified just anterior to the left diverticulum. Bilateral diverticulectomies were performed. The right ureter was reimplanted without a tunnel and an externalized stent was placed. Since the left ureter was not involved in the left diverticulum it was not reimplanted. Inspection of the bladder neck showed an abnormal trigone with laterally displaced ureteral orifices bilaterally and a hypoplastic bladder neck. No discreet urethra was noted and a feeding tube could not be passed into the urethra via a antegrade fashion. Next the urachus was excised. The bladder defects were closed in one layer with absorbable suture. The bladder specimen was then sent to pathology for a histology review (Figure3). A structure adherent to the posterior aspect of the bladder was identified as the uterus with an atretic vaginal canal and no obvious vaginal orifice. A right fallopian tube and ovary were identified but no left fallopian tube or gonad was found. A cutaneous vesicostomy was created for maximal bladder drainage.

Postoperatively, the patient’s creatinine stabilized at 0.6 mg/dl by day 19 of life. Ultrasonography at this time demonstrated stable hydronephrosis bilaterally. The patient did well in the immediate postoperative period with mechanical ventilation and enteral feeds. However, she was unable to be weaned from the ventilatory over the ensuing months. Six months later the patient expired from overwhelming sepsis secondary to hospital acquired pneumonia.

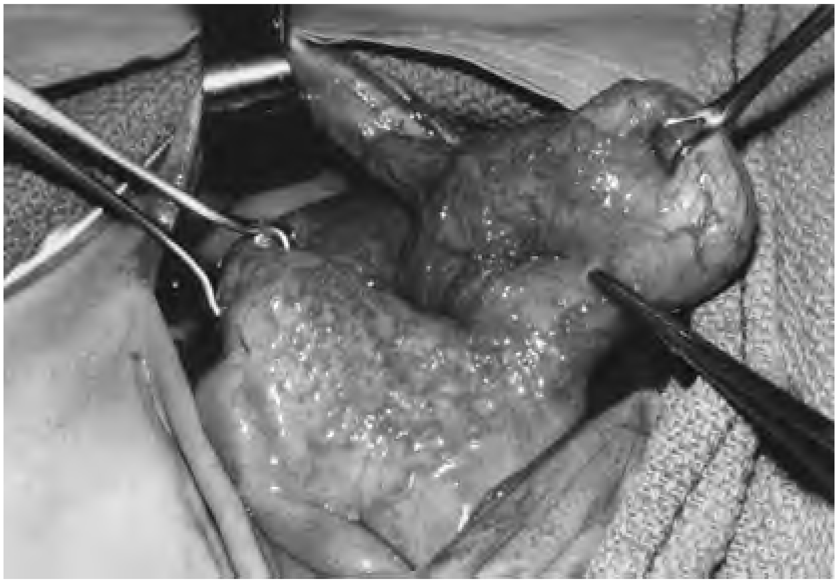

**FIGURE 2.** Gross presentation of the bladder demonstrating bladder diverticulum and urachus.

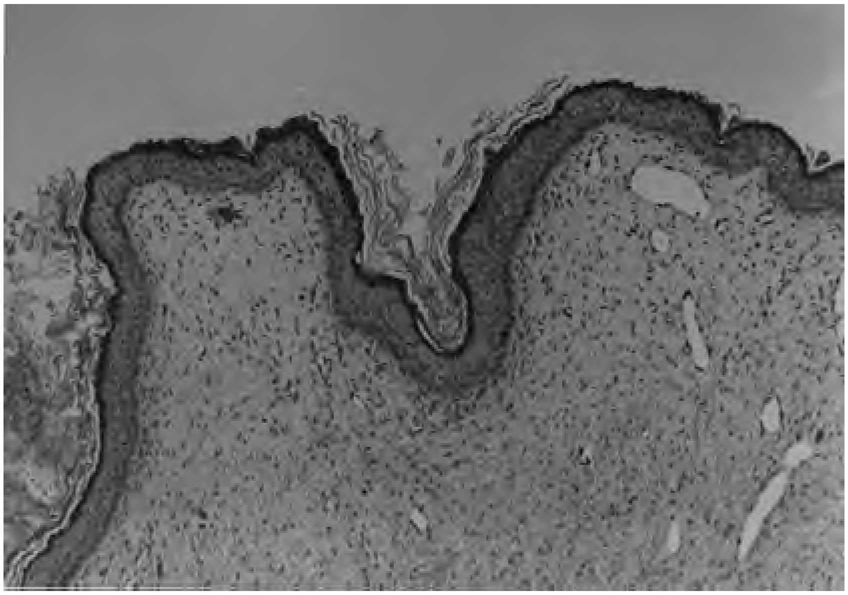

**FIGURE 3**. Microscopic examination of the bladder illustrating hypertrophy of the muscularis propia from chronic obstruction.

### DISCUSSION

The Prune Belly Syndrome in females is a rarer occurrence than in males with an observed prevalence in males of 5/100,000 versus 1/100,000 in females. By definition this syndrome is only completely expressed in males(presence of cryptorchidism mandatory), however, three percent of the reported cases occur in genetic females and is generally known as Pseudo-Prune Belly Syndrome(PPBS).[4,5] Our patient was found to have the typical phenotype of PPBS, lax abdominal musculature, large distended bladder, and urethral atresia as well as an unilateral absent ovarian structure, absent left fallopian tube and atretic vagina. These anomalies are consistent with other reported cases of PPBS in which vaginal atresia, bicornuate uterus, urethral atresia, and hydronephrosis are commonly noted.[6]

A detailed search of the literature revealed no reported association between the PPBS and tracheo-esophageal fistula. It is interesting to note that both PPBS and tracheo-esophageal fistula in associated with VATER syndrome are described with the same type of urinary tract anomaly, hydroureteronephrosis. Lloyd et. al did speculate that tracheo-esophageal in association with VATER syndrome is due to a teratogenic insult between 32-49 days of gestation, a similar time period for the development of PPBS after exposure to an unidentified teratogen.[8] This would argue for the possibility of an insult occurring during development that resulted in the concomitant defects seen in this patient. However, our patient did not have any other characteristics of VATER syndrome. The distal tracheo-esophageal fistula with a proximal pouch is the most common type of tracheo-esophageal. It is suspected that the gastric perforation was the result of barotraumas from the mechanical ventilation.

Reinberg reported the only other case of Prune Belly Syndrome, VATER syndrome, and urethral atresia in a stillborn and stated that the urethral atresia without a patent urachus or vesicocutaneous fistula is a universally lethal anomaly.[3] However, our patient had no patent urachus or vesicocutaneous fistula. She did, however, receive weekly ultrasound guided needle drainage of her bladder after 19 weeks of gestation. Fluid from the bladder drainage was not sent for analysis. The bladder drainage performed in utero to relieve the obstructive process affecting the urinary tract may have improved the neonatal outcome by reducing the intra-abdominal pressure and volume and thus avoiding pulmonary maldevelopment. The effect of this bladder drainage on the ultimate renal function is less clear.

The vesico-amniotic shunt was inserted and its correct position confirmed by ultrasonography. However possible due to the twin pregnancy and monochorionic gestation, the vesico-amniotic shunt was noted to have migrated 1 week after its placement. This is consistent with previous reports which state that approximately one third of shunts placed will migrate and become non-functional. As for the pre-term labor, it can not be ruled out that it was not due to the multiple percutaneous bladder drainage procedure. However gross and microscopic inspection of the placenta after birth did not reveal any evidence of chorioamnionitis.

Postnatally, the patient creatinine of 0.3 mg/dl reflected the mother's ability to filter toxins while the patient was in utero. However by day4 of life, it was evident that the patient's hydroureteronephrosis had worsen as was evident by ultrasonography and a rising creatinine of1.0 mg/dl. After maximal urinary drainage was provided via a cutaneous vesicostomy, the patient's creatinine returned to 0.6 mg/dl. Duckett stated if the nadir creatinine level is less than 0.8 mg/dl, the patient's chance of developing renal failure is dramatically less.[9] Postoperative ultrasonography demonstrated stable bilateral hydronephrosis.

Pediatric urologists and pediatricians should be aware of the potential for other anomalies when confronting a newborn with Prune Belly Syndrome.

### REFERENCES

1. Woodhouse, C.R., Ransley, P.G., Innes-Williams, D.: Prune Belly Syndrome—Report of 47 Cases. Archives of Disease in Childhood, 57 (11): 856-859, 1982.
2. Coran, A.G., Oldham, K.T.: The Pediatric Thorax. In Greenfield, L.J., Mulholland, M.W., Oldham, K.T., and Zelenock, G.B. Surgery Scientific Principles and Practices. J.B. Lippincott Company, 1993, pp. 1806-1831.
3. Reinberg, Y., Chelimsky, G., and Gonzalez, R.: Urethral Atresia and the Prune Belly Syndrome. Report of Six Cases. British Journal of Urology, 72 (1): 112-114, 1993.
4. Donnelly, L.F., Johnson, J.F.: Unilateral Abdominal Wall Hypoplasia: Radiographic Findings in Two Infant Girls. Pediatric Radiology, 25 (4): 278-281, 1995.
5. Druschel, C.M.: A Descriptive Study of Prune Belly Syndrome in New York State, 1983-1989. Archives of Pediatric Adolescent Medicine, 149: 70-76, 1995.
6. Reinberg, Y., Shapiro, E., Manivel, C.J., Manley, C.B., Pettinato, G., and Gonzalez, R.: Prune Belly Syndrome in Females: A Triad of Abdominal Muscular Defiency and anomalies of the Urinary and Genital Systems. The Journal of Pediatrics, 118 (3): 395-398, 1991.
7. Sutherland, R.S., Mevorach, R.A., and Kogan, B.A.: The Prune Belly Syndrome: Current Insights. Pediatric Nephrology, 9: 770-778, 1995.

8. Lloyd, D.J., Mckenzie, J., Kaye, H.H., and Russell, G.: VATER Syndrome: Hypothesis and Report of Two Further Cases. Teratology, 15: 43-46, 1976.
9. Coplen, D.E., Snow, B.W., Duckett, J.W.: Prune Belly syndrome. In Gillenwater, J.Y., Grayhack, J.T., Howards, S.S., Duckett, J.W. Adult and Pediatric Urology, ed 3. St. Louis, Mosby, 1996 , pp 2297-2316.

**This article should be referenced as follows:**

Potter, L., Rosser, C.J., Iskandar, S., and Kroovand, R.L. (2004) Prune Belly Syndrome and tracheo-esophageal fistula in a premature neonate. *TheScientificWorldJOURNAL* **4 (S1)**, 291–295.

**Handling Editor:**

Anthony Atala, Principle Editor for *Urology* — a domain of *TheScientificWorldJOURNAL*.
